# Human bone marrow niche chemoprotection mediated by cytochrome p450 enzymes

**DOI:** 10.18632/oncotarget.3614

**Published:** 2015-04-10

**Authors:** Salvador Alonso, Meng Su, Jace W. Jones, Sudipto Ganguly, Maureen A. Kane, Richard J. Jones, Gabriel Ghiaur

**Affiliations:** ^1^ Sidney Kimmel Comprehensive Cancer Center, Department of Oncology, Johns Hopkins University, Baltimore, MD, USA; ^2^ Department of Pharmaceutical Sciences, University of Maryland School of Pharmacy, Baltimore, MD, USA

**Keywords:** drug resistance, leukemia, microenvironment, CYP, multiple myeloma

## Abstract

Substantial evidence now demonstrates that interactions between the tumor microenvironment and malignant cells are a critical component of clinical drug resistance. However, the mechanisms responsible for microenvironment-mediated chemoprotection remain unclear. We showed that bone marrow (BM) stromal cytochrome P450 (CYP)26 enzymes protect normal hematopoietic stem cells (HSCs) from the pro-differentiation effects of retinoic acid. Here, we investigated if stromal expression of CYPs is a general mechanism of chemoprotection. We found that similar to human hepatocytes, human BM-derived stromal cells expressed a variety of drug-metabolizing enzymes. CYP3A4, the liver's major drug-metabolizing enzyme, was at least partially responsible for BM stroma's ability to protect multiple myeloma (MM) and leukemia cells from bortezomib and etoposide, respectively, both *in vitro* and *in vivo*. Moreover, clarithromycin overcame stromal-mediated MM resistance to dexamethasone, suggesting that CYP3A4 inhibition plays a role in its ability to augment the activity of lenalidomide and dexamethasone as part of the BiRd regimen. We uncovered a novel mechanism of microenvironment-mediated drug resistance, whereby the BM niche creates a sanctuary site from drugs. Targeting these sanctuaries holds promise for eliminating minimal residual tumor and improving cancer outcomes.

## INTRODUCTION

The majority of patients with acute myeloid leukemia (AML) and other hematologic malignancies achieve complete remissions with initial chemotherapy, but eventually relapse and die of their disease. The mechanisms responsible for the resistance of minimal residual disease (MRD) to therapy remain critical areas of research. There is increasing evidence that specialized microenvironments or niches play important roles in drug resistance [1–4]. Several studies have suggested a role for physical interactions as well as soluble factors in niche-mediated chemoprotection [5]. Accordingly, clinical trials targeting the microenvironment to improve chemosensitivity are ongoing [6, 7].

However, the exact mechanisms responsible for the chemoprotection afforded by the tumor microenvironment remain unclear. It is well recognized that specific tissues such as the central nervous system and the testes can act as sanctuary sites from drugs [8, 9]. We previously showed that bone marrow (BM) stroma protects normal human hematopoietic stem cells (HSCs) from the pro-differentiating effects of retinoic acid by expressing the retinoid-inactivating enzyme, CYP26 [10]. Here, we investigate if the BM microenvironment's expression of drug-metabolizing enzymes may represent a previously unrecognized more general mechanism of chemoprotection. We show that human BM stromal cells, but not hematopoietic cells, express drug-metabolizing enzymes at levels comparable to human hepatocytes. Moreover, we found that stromal CYP3A4 is at least partially responsible for microenvironment-dependent drug resistance.

## RESULTS

### Bone marrow stroma highly expresses drug metabolizing enzymes

**Figure 1:**
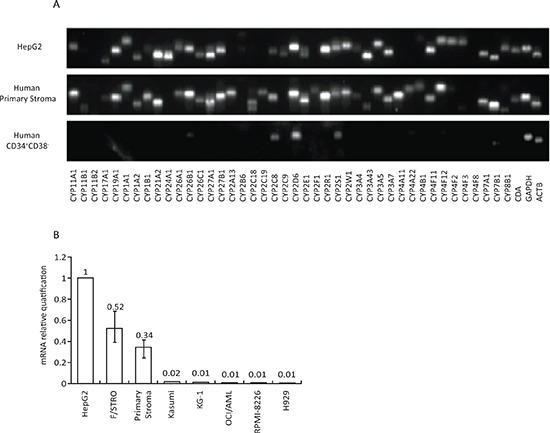
Expression of CYP enzymes in BM stroma cells **A.** RT-PCR analysis of mRNA expression of 42 CYP enzymes in the primary human BM stroma (middle panel) and human BM CD34^+^CD38^−^ HSC compartment (lower panel). Human hepatocellular carcinoma cell line HepG2 (upper panel) was used as a positive control for CYP enzyme expression. One representative BM stroma from three independent samples with similar results is shown. **B.** Relative quantification of CYP3A4 mRNA expression in human BM stroma from three different healthy BM donors, the BM stroma cell line F/STRO, three AML cell lines (Kasumi, OCI-AML and KG-1) and two MM cell lines (RPMI-8226 and H929). CYP3A4 expression was normalized to GAPDH, and relative quantification was calculated using ΔΔCT. Expression of CYP3A4 is presented relative to HepG2. Results show mean ± SEM of 3 independent experiments. CDA – cytidine deaminase, ACTB – β-actin.

The CYP superfamily encompasses 57 enzymes that catalyze the oxidation of various organic substrates, from intermediary steps in cholesterol metabolism to drugs and other chemicals [13]. Most of the xenobiotic and drug metabolizing CYPs are expressed in the liver. Extra-hepatic expression is usually restricted to barrier tissue such as lungs, skin, and gut [14]. Using qRT-PCR array, a total of 42 CYPs were analyzed in primary human BM stroma. Most enzymes displayed comparable mRNA levels in stroma and HepG2 cells, a hepatocellular carcinoma cell line routinely used for study of CYP enzymes [15] (Figure 1A and Supplementary Table). In contrast, the human HSC compartment (CD34^+^CD38^−^) showed limited expression of CYPs, with only low relative mRNA levels of two drug-metabolizing CYPs (2C8 and 2D6) and the orphan 2S1. Cytidine deaminase, another important drug metabolizing enzyme showed the same pattern of expression (Figure 1A).

The expression of CYP3A4, estimated to metabolize more than half of all chemotherapeutics [16], was further confirmed in primary BM stroma cells as well as human BM mesenchymal progenitor cells, F/STRO, by qRT-PCR. We found that CYP3A4 mRNA expression was similar in F/STRO and human primary BM stroma. CYP3A4 levels in BM stroma were about 30–50% of the levels in HepG2 cells, whereas its expression was barely detectable in acute myeloid leukemia (AML) (Kasumi 1, OCI-AML3 and KG1) and multiple myeloma (MM) (H929 and RPMI 8226) cells (Figure 1B).

### CYP3A4 inhibition reverses stromal-mediated MM resistance to chemotherapy

**Figure 2:**
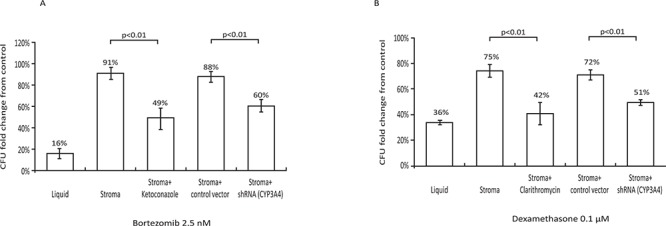
Role of stromal CYP3A4 in stroma-mediated resistance of MM cells to chemotherapy **A.** Effects of bortezomib (2.5 nM for 72 h) on the clonogenic recovery of H929 MM cells. Treatment of H929 cells with bortezomib resulted in only 16 ± 3% clonogenic recovery compared to control untreated cultures (first column, *p* < 0.01 vs control). BM stroma provided protection from bortezomib (second column). Concomitant treatment with 3 μM ketoconazole partially restored the sensitivity of H929 cells to bortezomib (third column, *p* < 0.01 stroma + bortezomib + ketoconazole vs. stroma + bortezomib). CYP3A4 knockdown by shRNA similarly restored the sensitivity of H929 cells to bortezomib (fifth column, *p* < 0.01, CYP3A4 knockdown stroma vs. control-infected stroma). **B.** Effects of dexamethasone (0.1 μM for 72 h) on the clonogenic recovery of H929 MM cells. Treatment of H929 cells with dexamethasone resulted in 36 ± 2% clonogenic recovery compared to control untreated cultures (first column, *p* < 0.01 vs control). BM stroma provided protection from dexamethasone (second column). Concomitant treatment with 1 μM clarithromycin partially restored the sensitivity of H929 cells to dexamethasone (third column, *p* < 0.01 stroma + dexamethasone + clarithromycin vs. stroma + dexamethasone). CYP3A4 knockdown by shRNA similarly restored the sensitivity of H929 cells to dexamethasone (fifth column, *p* < 0.01, CYP3A4 knockdown stroma vs. control-infected stroma). Data are presented as mean ± SEM of at least three independent experiments.

As previously reported, MM cells are highly resistant to bortezomib in the presence of BM stroma [2] (Figure 2A). Since bortezomib is predominantly metabolized by CYP3A4, we inhibited this enzyme in stroma by either ketoconazole or by lentiviral-mediated knockdown via shRNA (this method effectively eliminated CYP3A4 mRNA from stroma cells - Supplementary Figure 1). Both approaches partially restored bortezomib's activity against MM cells in the presence of BM stroma (Figure 2A). Ketoconazole had no effect in the absence of BM stroma (data not shown).

The addition of clarithromycin (Biaxin) to lenalidomide and dexamethasone has been recently shown to improve remission rates not only in treatment-naïve patients [17, 18] but also patients previously resistant to lenalidomide and dexamethasone [19]. While an initial report suggested that clarithromycin might have single agent activity in MM [20], subsequent data demonstrated no activity as a single agent [21, 22]. Since clarithromycin is a potent CYP3A4 inhibitor [23, 24], we investigated if this may be a potential mechanism for its activity in MM as part of BiRd [Biaxin, lenalidomide (Revlimid), dexamethasone] regimen. BM stroma was incubated with 10^−6^ M dexamethasone and drug levels were measured by liquid chromatography – tandem mass spectrometry (LC-MS/MS) (Supplementary Figure 2). After 24 h, dexamethasone levels were 70% reduced in the stromal conditioned media compared to liquid control (*p* < 0.01). Clarithromycin or anti - CYP3A4 shRNA restored the dexamethasone levels in the stromal conditioned media.

Accordingly, inhibition of CYP3A4 by clarithromycin or shRNA-mediated knockdown reversed the stromal-mediated protection of MM cells against dexamethasone (Figure 2B). Clarithromycin had no effect on dexamethasone's anti-MM activity in the absence of mesenchymal stroma (data not shown).

### CYP3A4 inhibition reverses stromal-mediated AML resistance to chemotherapy

**Figure 3:**
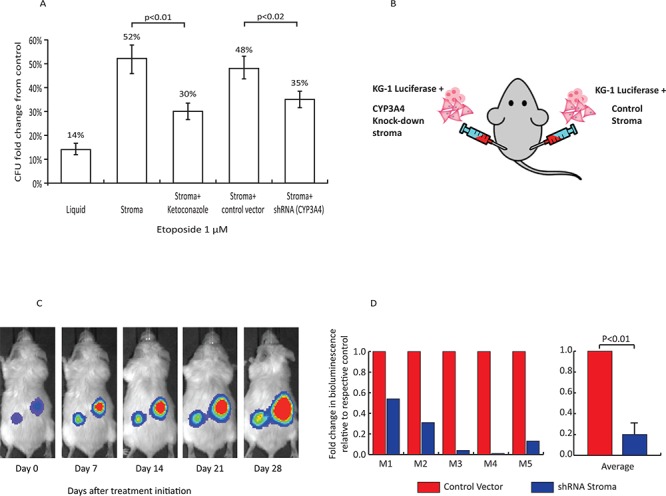
Role of stromal CYP3A4 in stroma-mediated resistance of AML cells to chemotherapy **A.** Effects of etoposide (1 μM for 72 h) on clonogenic activity of KG1 AML cells. Treatment of KG1 with etoposide resulted in 14 ± 2% clonogenic activity compared to control cultures, (first column, *p* < 0.01). BM stroma provided protection from etoposide (second column). Treatment with 3 μM ketoconazole rescued the protective effect seen in stroma co-culture (third column, *p* < 0.01, stroma + etoposide + ketoconazole vs. stroma + etoposide). CYP3A4 knockdown by shRNA rescued the sensitivity of KG1 cells to etoposide (fifth column, *p* < 0.02, CYP3A4 knockdown stroma vs. control-infected stroma). **B.** Xenograft model. Tumors on the left flanks consisted of a combination of KG1 luciferase + cells and CYP3A4-knockdown BM stroma cells. On the right flank, tumors consisted of KG1 luciferase + cells and stroma cells transduced with a control vector. **C.** Bioluminescent images of a representative xenograft mouse showing tumor burden during 4 weeks of treatment. **D.** Left panel depicts bioluminescent intensity (photons/second) for individual mice (M1-M5). The change in bioluminescence for each shRNA tumor at day 1 was normalized to the change in bioluminescence for each control tumor. Right panel shows the average ± SEM of 5 independent xenografts.

The topoisomerase inhibitor etoposide, which is also predominantly metabolized by CYP3A4 [25], remains one of the most important agents in the treatment of AML. BM stroma protected AML cells from the cytotoxic effects of etoposide (Figure 3A). Similar to our findings with bortezomib and dexamethasone, inhibition of CYP3A4 by ketoconazole or shRNA CYP3A4 knockdown partially restored etoposide cytotoxicity in the presence of stroma (Figure 3A).

To further study the role of microenvironment drug detoxifying enzymes in an *in vivo* model, we developed a xenograft model of human AML - BM stroma interactions. Luciferase expressing KG-1 AML cells and human BM stroma transduced with either control shRNA (Control) or anti-CYP3A4 shRNA (CYP3A4 KO) were injected subcutaneously into NSG mice (Figure 3B). Each mouse carried AML cells + Control stroma in the right flank and AML + CYP3A4 KO stroma in the left flank. After establishment of the tumors (14 days post subcutaneous injection), the mice were treated with etoposide (1.2 mg/kg/day) by intraperitoneal injection. There was no difference in tumor growth before initiation of etoposide (Supplementary Figure 3A). However, while the AML continued to grow exponentially with the Control stroma, the AML with CYP3A4KO stroma showed a significant response to etoposide treatment (Figure 3C and 3D and Supplementary Figure 3B).

## DISCUSSION

Newer chemotherapeutic agents have dramatically improved the response rates of hematological malignancies, yet progress toward cures has lagged. Research efforts have identified unique cell-intrinsic [26], and more recently, tumor microenvironment-dependent, cell-extrinsic mechanisms of drug resistance [6]. Although agents targeting the malignant microenvironment are currently in various stages of clinical development [6]. The mechanisms responsible for microenvironment-mediated drug resistance have remained unclear and are likely multifactorial. Several studies have suggested a role for physical interactions mediated by adhesion molecules. Accordingly, treatment with cytarabine in combination with a blocking antibody to integrin α_4_β_1_, and thus impaired adhesion to fibronectin, resulted in improved disease-free survival in a murine model of AML [27]. Similarly, the combination of bortezomib and the CXCR4 antagonist AMD3100 partially overcame microenvironment-mediated resistance in MM [28]. Soluble factors may also participate in microenvironment-mediated chemoprotection, as stromal co-culture enhanced malignant cell drug resistance in the absence of physical interactions [1, 29]. However, monoclonal antibodies targeting potential etiologic factors and cytokines such as interleukin-6 (IL-6), vascular endothelial growth factor (VEGF), and basic fibroblast growth factor (bFGF), did not reverse stroma-mediated resistance [1].

Although CYP3A4 and other CYP enzymes have been implicated in both systemic (by hepatic inactivation) [30–33] and cell-intrinsic [34, 35] drug resistance, their role within the tumor microenvironment has never been studied. Here, we show for the first time that CYP enzymes are highly expressed in BM stroma. Moreover, CYP3A4 expression appears to participate in the chemoprotection provided by BM stroma against both MM and AML. Other drug metabolizing enzymes, such as cytidine deaminase which is responsible for cytarabine metabolism, are also expressed by BM stroma. Thus, drug metabolizing enzymes expressed in the tumor microenvironment could represent biochemical barriers between plasma and unique malignant cell niches resulting in potential sanctuary sites from drugs. Pharmacokinetic parameters such as affinity (Km) and velocity (Vmax) constants may allow CYPs to play complementary roles in the systemic (hepatic) and local (microenvironmental) inactivation of drugs [36].

While initial studies hypothesized that clarithromycin may work in MM via its immunomodulatory effects [37], our results suggest that it augments the cytotoxicity of dexamethasone via inhibition of stromal CYP3A4. Not only does charithromycin overcome MM resistance to lenalidomide and dexamethasone [19], but it also increases response rates when used as upfront therapy [18]. These data together with our results suggest that the addition of clarithromycin could preferentially target a subset of MM cells not usually affected by lenalidomide and dexamethasone, perhaps those in close contact with the BM microenvironment.

Targeting drug metabolizing enzymes in the tumor microenvironment holds promise for eliminating MRD and improving cancer outcomes. Inhibition of CYPs in the liver has been studied clinically, with expected increased systemic drug levels. Pharmacologically adjusting drug doses to maintain safe systemic concentrations in the presence of CYP inhibitors, should control for hepatic inhibition of these enzymes while at the same time removing the barriers to therapeutic drug levels in the tumor microenvironment.

## MATERIALS AND METHODS

### Cell cultures

All cell lines except F/STRO were purchased from American Type Culture Collection and cultured per their recommendations. F/STRO was a kind gift from Dr. Pierre Marie; and was cultured as previously described [11]. Primary human BM stroma and hematopoietic cells were derived from aspirates collected from normal donors on an IRB-approved protocol at Johns Hopkins as we previously described [10].

For co-cultures, stromal cells were irradiated (20Gy) and plated to generate a monolayer. Hematopoietic cells were plated on top of the monolayer and treated with 2.5 nM bortezomib (Millennium Pharmaceuticals, Cambridge, MA), 1 μM etoposide (Sigma, St. Louis MO), 0.1 μM dexamethasone (Sigma) ± 3 μM ketoconazole (Sigma) or 1 μM clarithromycin (Sigma) for 72 h at 37°C.

### Clonogenic assays

Clonogenic assays were performed as described [10]. Briefly, after drug incubations, the malignant cells were collected, washed with PBS and plated (700 cells/ml for KG1 and 5000 cells/ml for H929) in 1 ml of 1.32% methylcellulose (Sigma) supplemented with 30% FBS, 1% bovine serum albumin (BSA – Sigma), 2 mM L-glutamine and 0.1 μM 2-ME. Cells were plated in triplicates in 35 mm culture dishes, incubated at 37°C and scored for the presence of colonies at day 10 for KG-1 and day 14 for H929 cells.

### Lentiviral infection: shRNA and luciferase

Lentiviral pLKO.1shRNA vectors were obtained from the RNAiConsortium (Broad Institute, Cambridge, MA). Supernatants were produced and stromal cells were infected as we have previously published [10]. Infected cells were selected using 3 μg/ml of puromycin (Sigma) for 5 days. To mark KG-1 cells with Luciferase, pLenti-CMV-LUC-Puro lentiviral vectors [12] were purchased from Addgene (plasmid #17477) and lentiviral supernatants were produced as we have previously published [10]. KG-1 cells were incubated with the lentiviral supernatant in the presence of 8 μg/mL of polybrene (Sigma) and span down at 2500 RPM, 30 min at room temperature. After centrifugation, the cells were cultured at 37ºC for at least 48 h prior to selection using 1 μg/mL puromycin.

### Quantitative reverse transcriptase-polymerase chain reaction (qRT-PCR)

Total RNA was extracted using the RNeasy Mini Kit (QIAGEN, Valencia, CA). cDNA was synthesized by reverse transcription using the iScript cDNA synthesis kit (BIO-RAD, Hercules, CA). Quantitative RT-PCR was performed with iTaq SYBR Green Supermix (BIO-RAD) as we have published [10]. Quantitative RT-PCR for 42 different CYPs was performed using the Drug Metabolism Phase I Enzymes PCR Array (QIAGEN) per manufacturer's protocol.

### Xenograft mouse model

Five hundred thousand KG-1 Luciferase (+) cells and 2.5 × 10^5^ primary human BM stroma cells previously infected with either control lentivirus (Control stroma) or anti CYP3A4 shRNA lentivirus (CYP3A4 knockdown stroma) were injected in each flank (Control stroma in right flank, CYP3A4 knockdown stroma in left flank) of 16-week old of NOD SCID IL2γ^−/−^ (NSG) mice (The Jackson Laboratory, Bar Harbor, ME). After tumor engraftment, as determined by exponential increase in bioluminescence, mice were treated with 1.2 mg/kg etoposide via daily intraperitoneal injection. Tumor burden was assessed by bioluminescence, using the *In Vivo* Imaging System (IVIS, Perkin Elmer, Alameda, Ca). For imaging, mice were exposed to 30 mg/kg D-luciferin (Xenogen) via intraperitoneal injection 10–15 minutes prior to imaging and were anesthetized using isoflurane (VetOne, Boise, ID). Images were analyzed with Living Image Software 2.5 (Perkin Elmer) and data quantified as photons/second.

### Quantification of dexamethasone

Complete media was supplemented with dexamethasone (10^−6^ M) (Sigma) and incubated at 37°C either in the absence of stroma or in the presence of stroma or shRNA mediated CYP3A4 knock downed stroma with or without 10^−6^ M clarithromycin (Sigma). Dexamethasone was quantified 24 h later by liquid chromatography-tandem mass spectrometry (LC-MS/MS) on a TSQ Quantum Ultra Triple Stage Quadrupole Mass Spectrometer coupled to an Ultimate 3000 RS Liquid Chromatogram system (Thermo Scientific, San Jose, CA).

### Statistical analysis

Statistical significance was evaluated using 2-tailed unpaired student *t* test.

## SUPPLEMENTARY MATERIALS FIGURES AND TABLE


