# Supplementary material for: Human bone marrow niche chemoprotection mediated by cytochrome p450 enzymes
Source: Oncotarget. 2015 Apr 10;6(17):14905–12. doi: 10.18632/oncotarget.3614 (PMC4558124; doi:10.18632/oncotarget.3614)
Supplement: Supplementary file 1 [file oncotarget-06-14905-s001.pdf]

SUPPLEMENTARY MATERIALS

COMPLETE METHODS

Cell cultures

The MM cell line H929 and the AML cell line KG-1 were purchased from the American Type Culture Collection (ATCC). Both cell lines were cultured at 37°C in RPMI 1640 medium (Sigma, St. Louis MO) supplemented with 10% fetal bovine serum (FBS – Sigma), L-glutamine (Sigma) and penicillin/streptomycin (P/S - Sigma). The human BM-derived stroma cell line F/STRO was a kind gift from Dr. Pierre Marie; it was cultured at 33°C in DMEM medium (Sigma) supplemented with 10% FBS, L-glutamine and P/S as previously described(1). HepG2, a hepatocellular carcinoma cell line was also purchased from ATCC and cultured in αMEM supplemented with 10% FBS, L-glutamine and P/S.

Primary human BM stroma cells and hematopoietic cells were derived from BM aspirates collected from normal donors for allogeneic transplantation on an IRB-approved protocol at Johns Hopkins University. Adherent BM stromal cultures were performed as we have recently published(2). Briefly, BM mononuclear cells (MNCs) were isolated by density gradient centrifugation, then cultured in IMDM media supplemented with 5% horse serum (Sigma), 15% FBS, 10 µM hydrocortisone (Sigma), P/S and 0.1 µM β-mercaptoethanol (2-ME, Sigma) at 33°C, until an adherent confluent monolayer was obtained. The cells were passaged upon washing with phosphate buffered saline (PBS - Sigma) and enzymatic treatment with Trypsin (Sigma), and each passage was recorded. Experiments presented in this study were performed with early passage (less than 4) human primary BM stroma cells.

$CD34^{+}CD38^{-}$ cells were also isolated from normal donor BM MNCs and sorted as we have previously published(2). Briefly, $CD34^{+}$ cells were selected using magnetic beads and column isolation (Miltenyi Biotec, Cambridge, MA). These cells were then labeled with fluorescein isothiocyanate (FITC) and phycoerythrin (PE) conjugated monoclonal anti-CD34 and anti-CD38 (BD biosciences, San Jose, CA), respectively, and sorted via FACS Aria II (BD biosciences).

For stromal co-cultures, F/STRO cells were irradiated (20Gy) and cultured for 24 h to generate a confluent monolayer by plating 2×10^{5} cells in a 24 well plate. At that time, H929 and KG1 cells were plated, either in liquid culture or on top of the stromal monolayer. Cells were then treated with 2.5 nM bortezomib (Millennium Pharmaceuticals, Cambridge, MA) or 1 µM etoposide (Sigma), in the presence or absence of 3 µM ketoconazole (Sigma), and incubated for 72 h at 37°C.

Clonogenic assays

Clonogenic assays were performed as previously described(2). Briefly, after drug incubations, the malignant cells were collected, washed with PBS and plated (700 cells/ml for KG1 and 5000 cells/ml for H929) in 1ml of 1.32% methylcellulose (Sigma) supplemented with 30% FBS, 1% bovine serum albumin (BSA – Sigma), 2mM L-glutamine and 0.1 µM 2-ME . Cells were plated in triplicates in 35mm culture dishes, incubated at 37°C and scored for the presence of colonies at day 10 for KG-1 and day 14 for H929 cells.

Lentiviral infection: shRNA and luciferase

Lentiviral pLKO.1 shRNA vectors were obtained from the RNAi Consortium (Broad Institute, Cambridge, MA). Lentiviral supernatants were produced as we have previously published(2). Briefly, 293T packaging cells (ATCC) were transfected with a lentivirus vector encoding shRNA targeted to CYP3A4 or an empty vector together with pCMV-dR8.9 (Δ8.9) and VSV-G expressing plasmids and incubated for 48 h. The supernatant was collected, filtered through a 0.45 µM filter and stored at -80°C until ready to use. For transductions, F/STRO cells were incubated with the viral supernatant in the presence of 8 µg/mL polybrene (Sigma) for 12 h. Infected cells were selected using 3 µg/ml of puromycin (Sigma) for 5 days and were maintained in the presence of puromycin until further use.

To mark KG-1 cells with Luciferase, pLenti-CMV-LUC-Puro lentiviral vectors[12] were purchased from Addgene (plasmid #17477) and lentiviral supernatants were produced as we have previously published[10]. KG-1 cells were incubated with the lentiviral supernatant in the presence of 8 µg/mL of polybrene (Sigma) and span down at 2500 RPM, 30 min at room temperature. After centrifugation, the cells were cultured at 37ºC for at least 48 h prior to selection using 1 µg/mL puromycin.

Quantitative reverse transcriptase-polymerase chain reaction (RT-PCR)

Total RNA was extracted using the RNeasy Mini Kit (QIAGEN, Valencia, CA). cDNA was synthesized by reverse transcription using the iScript cDNA synthesis kit (BIO-RAD, Hercules, CA). Quantitative RT-PCR (qPCR) was performed with iTaq SYBR Green Supermix (BIO-RAD) and run on the ABI 7900 system (Life Technologies, Grand Island, NY). CYP3A4 mRNA expression was normalized to GAPDH, and relative quantification was calculated using ΔΔ CT as we have previously published(2).

PCR array

Quantitative RT-PCR for 42 different P450 enzymes was performed using the Drug Metabolism Phase I Enzymes PCR Array (QIAGEN) per manufacturer's protocol. Briefly, total RNA was extracted and transcribed to cDNA as previously described. RT-PCR was run on the StepOne Plus machine (BD biosciences). Enzyme mRNA relative quantification was calculated using ΔΔCT, normalized to GAPDH expression as we previously described(2).

Xenograft mouse model

Fifty thousand KG-1 Luciferase (+) cells and 2.5×10^4 primary human BM stroma cells previously infected with either control lentivirus (Control stroma) or anti CYP3A4 shRNA lentivirus (CYP3A4 knockdown stroma) were injected in each flank (Control stroma in right flank, CYP3A4 knockdown stroma in left flank) of 16-week old of NOD SCID IL2$\gamma^{-/-}$ (NSG) mice (The Jackson Laboratory, Bar Harbor, ME). After tumor engraftment, as determined by exponential increase in bioluminescence, mice were treated with 1.2 mg/kg etoposide via daily intraperitoneal injection. Tumor burden was assessed by bioluminescence, using the *In Vivo* Imaging System (IVIS, Perkin Elmer, Alameda, Ca). For imaging, mice were exposed to 30 mg/kg D-luciferin (Xenogen) via intraperitoneal injection 10–15 minutes prior to imaging and were anesthetized using isoflurane (VetOne, Boise, ID). Images were analyzed with Living Image Software 2.5 (Perkin Elmer) and data quantified as photons/second.

Quantification of dexamethasone

Complete media was supplemented with dexamethasone (10^{-6} M) (Sigma) and incubated at 37°C either in the absence of stroma or in the presence of stroma or shRNA mediated CYP3A4 knock downed stroma with or without 10^{-6} M clarithromycin (Sigma). Dexamethasone was quantified by liquid chromatography-tandem mass spectrometry (LC-MS/MS). Dexamethasone and flumethasone were purchased from Sigma Aldrich (St. Louis, MO). Flumethasone was used as the internal standard. Optima LC/MS grade water, acetonitrile, ammonium formate, and formic acid were purchased from Fisher Scientific (Pittsburg, PA). All chemicals and reagents were used without further purification. Stock solutions of dexamethasone and flumethasone were prepared in water:acetonitrile (1:1 v/v). Calibration standards ranging from 0.01 μM to 500 μM of Dexamethasone with 10 μM flumethasone were prepared. Independent quality control samples at three concentration levels (0.05, 5.0, and 100 μM) were also prepared. Experimental samples were prepared as follows: 100 μL of sample was combined with 10 μM of flumethasone and 500 μL of acetonitrile for protein precipitation. The mixture was thoroughly mixed for 30 seconds followed by centrifugation at 10, 000 rpm for 10 minutes. 500 μL of supernatant was transferred and dried under a steady stream of nitrogen. The sample was re-suspended in 100 μL water:acetonitrile (1:1 v/v) for LC-MS/MS analysis. LC-MS/MS analysis was performed on a TSQ Quantum Ultra Triple Stage Quadrupole Mass Spectrometer coupled to an Ultimate 3000 RS Liquid Chromatogram system (Thermo Scientific, San Jose, CA). The liquid chromatography (LC) separation was performed on a Waters Cortecs C18+ (2.1 × 5 mm, 2.7 μm) (Waters, Milford, MA) equipped with a Cortecs C18+ Vanguard pre-column (2.1 × 100 mm, 2.7 μm) (Waters, Milford, MA) operated at 30°C. Solvent A and B consisted of 10 mM ammonium formate with 0.1% formic acid in water and acetonitrile, respectively. The gradient program was 0.0–0.5 min, 20% B; 0.5–2.5 min, gradient to 95% B; 2.5–4.0 min, 95% B; 4.0–4.5 min, gradient to 20% B; 4.5–6.0 min, 20% B. The flow rate was to 0.4 mL/min during all separation steps and injection volume was 10 μL. The tandem mass spectrometric (MS/MS) detection was performed in the negative-ion mode and the electrospray ionization (ESI) source parameters were as follows: spray voltage, 3000; capillary temperature, 300; sheath gas pressure, 30; ion sweep gas pressure, 0.2; capillary offset, 10; tube lens offset, 50–75. Selected reaction monitoring (SRM) was used for mass detection with the following transitions: Dexamethasone (*m/z* 437.2 → 307.1 and 360.9) and Flumethasone (*m/z* 455.2 → 305.0 and 379.1). The formate anion of both dexamethasone and flumethasone was used for SRM detection. Flumethasone was used as the internal standard. Data collection and analysis was performed by Xcalibur V 2.1 (Thermo Scientific, San Jose, CA) and Prism 6 (Graph Pad, La Jolla, CA).

Statistical analysis

Statistical significance was evaluated using 2-tailed unpaired student *t* test. All experiments were repeated a minimum of 3 times.

REFERENCES

1. Ahdjoudj S, Lasmoles F, Oyajobi BO, Lomri A, Delannoy P, Marie PJ. Reciprocal control of osteoblast/chondroblast and osteoblast/adipocyte differentiation of multipotential clonal human marrow stromal F/STRO-1(+) cells. J Cell Biochem. 2001; 81:23–38.
2. Ghiaur G, Yegnasubramanian S, Perkins B, Gucwa JL, Gerber JM, Jones RJ. Regulation of human hematopoietic stem cell self-renewal by the microenvironment's control of retinoic acid signaling. Proceedings of the National Academy of Sciences of the United States of America. 2013; 110:16121–6.

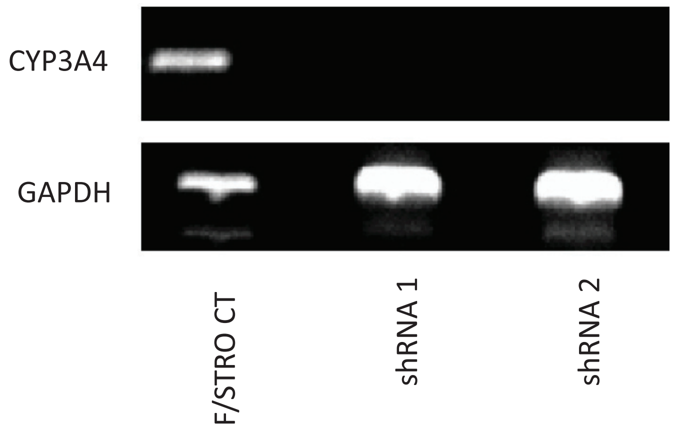

Supplementary Figure S1: Lentiviral-mediated shRNA-induced CYP3A4 knockdown. F/STRO human BM mesenchymal cells were infected with pLKO lentiviruses expressing two different shRNA targeting CYP3A4 (lines 2 and 3) or a control shRNA virus (first line). Upon infection, cells were selected in the presence of puromycin and RT– PCR was used to evaluate the presence of CYP3A4 mRNA. GAPDH was used as loading control. These cells were cultured in the presence of puromycin until used for experiments described in the manuscript.

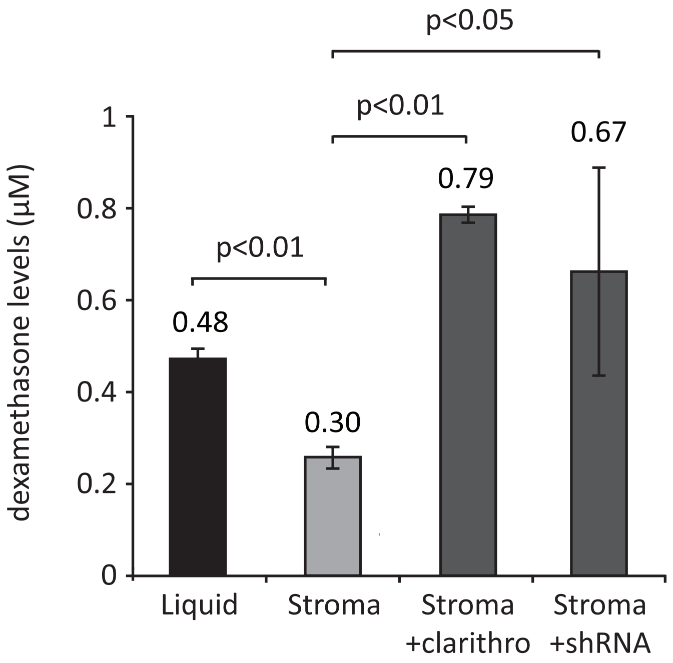

Supplementary Figure S2: Quantification of dexamethasone levels in BM stroma conditioned media. Complete media (RPMI + 10%FCS) was supplemented with 10^{-6} M dexamethasone and incubated at 37°C in a humidified incubator in 5% CO_2 either in the absence of human primary BM stroma (Liquid) or in the presence of human primary stroma that was previously unmanipulated (stroma) or infected with anti CYP3A4 shRNA (stroma + shRNA). Concomitent treatment with 10^{-6} M clarithromycin was used in stroma + clarithro condition. The concentration of dexamethasone was determined at 24 h. Data represent mean ± STD of three independent experiments.

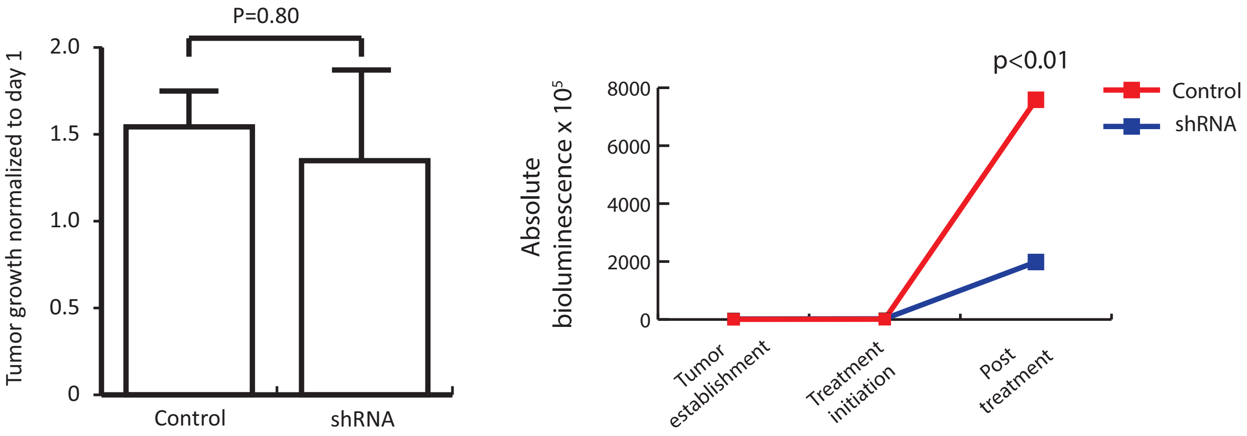

Supplementary Figure S3: Tumor burden during treatment with etoposide in KG1 + stroma xenografts. **A.** Bioluminescence of xenograft AML tumors that contain control shRNA infected stroma (Control) and anti-CYP3A4 shRNA infected stroma (shRNA) prior to treatment with etoposide. Data represent mean ± STD of fold change from day 0 of 5 xenograft mice. **B.** Absolute bioluminescence (photons/second × 10^5) of tumors with control or shRNA stroma at tumor establishment, chemotherapy initiation, and post-chemotherapy. Values represent average bioluminescence of each group ($n = 5$ mice per group) ($p < 0.01$, CYP3A4 knockdown stroma vs. control-infected stroma post-treatment).

Supplementary Table S1. Ct values of various CYP enzymes and cytidine deaminase (CDA)

	HepG2	Primary 1	Primary 2	Primary 3
CYP11A1	4.662	9.983	8.507	5.887
CYP11B1	14.444	17.051	14.746	17.215
CYP11B2	12.704	17.081	15.548	15.484
CYP17A1	7.912	17.167	15.386	12.930
CYP19A1	7.226	10.758	10.898	10.020
CYP1A1	0.651	14.786	14.928	12.596
CYP1A2	14.265	17.126	14.413	11.048
CYP1B1	14.255	2.063	0.943	-5.281
CYP21A2	7.229	12.971	12.812	12.473
CYP24A1	9.225	15.245	16.161	20.029
CYP26A1	12.233	14.986	15.746	13.020
CYP26B1	9.660	10.778	8.924	11.035
CYP26C1	14.342	15.592	16.027	17.020
CYP27A1	3.450	8.991	7.625	5.351
CYP27B1	8.668	12.974	12.393	13.857
CYP2A13	14.480	16.993	14.732	16.497
CYP2B6	11.732	16.027	16.123	15.883
CYP2C18	14.466	15.007	15.619	16.544
CYP2C19	ND	15.844	16.019	16.018
CYP2C8	14.365	14.969	15.708	14.027
CYP2C9	14.358	16.981	17.005	17.032
CYP2D6	8.018	11.979	10.735	12.030
CYP2E1	13.226	10.970	9.832	10.606
CYP2F1	14.480	16.159	16.032	17.193
CYP2R1	9.232	8.983	8.200	8.700
CYP2S1	7.237	14.983	14.756	6.161
CYP2W1	1.066	15.527	13.922	16.542
CYP3A4	12.920	15.946	15.953	16.768
CYP3A43	5.201	15.971	16.070	17.286
CYP3A5	7.624	13.037	14.907	12.032
CYP3A7	10.487	16.990	15.649	15.471
CYP4A11	14.494	15.977	13.466	14.999
CYP4A22	14.473	16.037	14.911	17.110
CYP4B1	8.602	14.987	12.405	12.148
CYP4F11	7.229	16.602	15.934	9.324
CYP4F12	-1.344	13.984	12.745	14.040
CYP4F2	5.244	16.500	16.110	17.160
CYP4F3	7.265	16.434	16.056	16.044
CYP4F8	ND	ND	ND	ND
CYP7A1	7.115	16.010	14.472	12.393

	HepG2	Primary 1	Primary 2	Primary 3
CYP7B1	13.236	8.002	5.925	4.194
CYP8B1	8.904	15.388	15.907	17.206
CDA	5.803	11.967	12.921	8.066
GAPDH CT	22.66	19.92	20.99	19.87714195

Primary 1, 2, 3 represent three independent human BM stroma. ND – not determined. GAPDH was used as internal control.
